# Differential MicroRNA Expression Pattern in Endothelial Progenitor Cells During Diabetic Retinopathy

**DOI:** 10.3389/fcell.2021.773050

**Published:** 2021-12-16

**Authors:** Ze-Hui Shi, Xiao-Yan Han, Mu-Di Yao, Chang Liu, Qin Jiang, Biao Yan

**Affiliations:** ^1^ Eye Institute, Eye & ENT Hospital, Shanghai Medical College, Fudan University, Shanghai, China; ^2^ The Affiliated Eye Hospital, Nanjing Medical University, Nanjing, China; ^3^ The Fourth School of Clinical Medicine, Nanjing Medical University, Nanjing, China; ^4^ NHC Key Laboratory of Myopia (Fudan University), Key Laboratory of Myopia, Chinese Academy of Medical Sciences, Shanghai Key Laboratory of Visual Impairment and Restoration (Fudan University), Shanghai, China

**Keywords:** microRNA, endothelial progenitor cells, diabetic retinopathy, vascular dysfunction, endothelial cell

## Abstract

Endothelial progenitor cells (EPCs) are involved in the pathogenesis of microvascular dysfunction in diabetic retinopathy (DR). MicroRNAs (miRNAs) serve as crucial regulators in many biological process and human diseases. Herein, to investigate the expression profile and possible role of miRNAs in EPCs, small RNA sequencing was conducted to identify EPC dysfunction-related miRNAs in DR. A total of 72 miRNAs were differentially expressed in EPCs following high glucose stress. Based on Gene Ontology (GO) analysis, the target genes of differentially expressed miRNAs were targeted to “protein binding,” “cell differentiation,” and “cytoskeleton.” Kyoto Encyclopedia of Genes and Genomes (KEGG) pathway analysis revealed that cGMP-PKG signaling pathway was tightly associated with miRNA-mediated EPC function. Furthermore, miR-375–3p was verified to be up-regulated in the clinical samples of DR patients. Inhibition of miR-375–3p protected against hyperglycemic stress- or hypoxic stress-induced EPC injury, which increased the viability, proliferation, migration, and tube formation ability of EPCs and retarded the development of apoptosis. Collectively, this study provides a novel insight into the pathogenesis of EPC dysfunction in DR. miR-375–3p is a potential target for the diagnosis or treatment of DR.

## Introduction

Microvascular dysfunction is a dominant complication caused by diabetes mellitus (DM). Endothelial injury has become the pathological basis of microvascular dysfunction (Cheung et al., 2010). Notably, diabetic retinopathy (DR), the most common microvascular complication of DM, is the leading cause of blindness among working-age people (Rodríguez et al., 2019). In DR, the breakdown of the blood–retinal barrier can damage endothelial cells, increase vascular permeability, and cause retinal ischemia and hypoxia (Semeraro et al., 2019). Although the existing treatments such as intravitreal pharmacologic agents (aflibercept, bevacizumab, ranibizumab, etc.) (Giurdanella et al., 2015), panretinal laser photocoagulation, and vitreous surgery can alleviate the progressive loss of visual function to a certain extent, the limitation of these treatments requires further studies to identify potential targets or new alternative therapies to prevent or delay the progression of DR (Heng et al., 2013).

As endothelial injury is the main pathological change of DR, inhibition of endothelial injury is critical for the prevention and treatment of DR. Previous studies have revealed that the injured endothelial cells can not only be repaired by the differentiated neighboring endothelial cells but also by endothelial progenitor cells (EPCs) (He et al., 2004; Wassmann et al., 2006). EPCs are mobilized from the bone marrow and migrate to the injured tissues, which contributes to endothelial cell repair (Zhang et al., 2002; Rana et al., 2018). EPCs have attracted great attention due to their critical roles in cardiovascular diseases, cerebral ischemia, cancers, and other diseases (Mandraffino and Saitta, 2018; Bayraktutan, 2019). Increasing evidence has suggested that the transplant of EPCs contributes to vessel repair (Wang et al., 2016; Ma et al., 2020). Both type 1 and type 2 diabetic patients have altered numbers of circulating EPCs. Clinical studies have revealed that circulating levels of CD34^+^ EPCs or CD133^+^/CD34^+^ EPCs increase in patients with DR when compared with the control group (Lee et al., 2006; Liu et al., 2010; Lois et al., 2014). The number and function of EPCs are changed in patients with different diabetes duration and metabolic control and in the presence or absence of DR (Zhang and Yan 2013; Shao et al., 2018). Thus, it is imperative to probe the underlying regulatory mechanism of EPC biology.

Accumulating evidence has revealed that non-coding RNAs play important roles in various physiological processes, including angiogenesis, cell differentiation, and metabolism (Krol et al., 2010; Beermann et al., 2016). MicroRNAs (miRNAs), a class of small non-coding RNA, are highly conserved and are 18–23 nucleotides in length. They have emerged as key regulators of gene expression by targeting the 3′-untranslated region (3′-UTR) of mRNAs, resulting in the degradation of target mRNA or reduction in protein translation (Krol et al., 2010). The abnormal expression of specific miRNAs is closely associated with the pathogenesis of human diseases, including cancers, cardiovascular diseases, and metabolic diseases (Landrier et al., 2019; Saliminejad et al., 2019). MiRNAs are also involved in the pathogenesis of DR by affecting endothelial cell differentiation, proliferation, and migration (Liu et al., 2018; Platania et al., 2019; Lu et al., 2020). However, the expression profiles and the underlying mechanisms of miRNA-mediated EPC biology are not fully understood.

In this study, we compared the expression pattern of miRNAs in bone marrow-derived EPCs with or without high glucose stress, and 72 miRNAs were differentially expressed between these two groups. Notably, miR-375–3p was found to be significantly up-regulated following high glucose stress. Functional assays indicated that miR-375–3p regulated the proliferation, apoptosis, migration, and tube formation of EPCs *in vitro*. This study provides a novel insight into the potential mechanism of EPC dysfunction under hyperglycemic condition.

## Materials and Methods

### Isolation and Culture of EPCs

EPCs were isolated from rat bone marrow and cultured as previously described (Zhang et al., 2014). Briefly, the bone marrow was separated from the femora and tibiae of SD rats (3 weeks old) under sterile conditions. Bone marrow mononuclear cells (BMMNCs) were further harvested by density gradient centrifugation (Histopaque 1,083, Sigma-Aldrich) at 400×*g* for 30 min at room temperature. The mononuclear cell layer was collected and washed with the complete EGM-2 medium (CC-3202, Lonza) three times. Then, EPCs were re-suspended with the complete EGM-2 medium and seeded in a six-well plate at a density of 8.5 × 10^5^ cell/cm^2^. They were incubated at 37 °C with 5% CO_2_ in humidified atmosphere. The culture medium was changed every 48 h. The non-adherent cells were removed after 3 days, and EPCs were used for the following experiments after a 7-days culture. EPCs were exposed to 30 mM d-glucose for 24 h to mimic a high-glucose condition. EPCs were treated with CoCl_2_ (200 μmol/L) for 24 h to mimic hypoxic stress.

### Identification of Bone Marrow-Derived Endothelial Progenitor Cells

The identification of EPC phenotypes was done by using fluorescence microscopy and flow cytometry. For the detection by fluorescence microscopy, both double-positive staining and immunofluorescent staining were performed. After 7 days of culture, EPCs were incubated with Dil-conjugated acetylated low-density lipoprotein (Dil-ac-LDL, L3484, Invitrogen) and Ulex Europaeus Agglutinin I (UEA-I, L9006, Sigma-Aldrich). Double-positive staining for Dil-ac-LDL and UEA-l was considered to represent EPCs and counted at × 40 magnification under a fluorescence microscope (IX73P1F, Olympus). For extracellular labeling of CD133, CD34, and VEGFR2, EPCs were fixed in 4% PFA for 30 min; blocked with 5% bovine serum albumin; and labeled sequentially with the antibodies against CD133, CD34, and VEGFR2, respectively (1:200, Santa Cruz Biotechnology). They were subsequently washed three times in PBS and counterstained with diamidino-phenylindole for 5 min. Following washing, the images were captured by fluorescence microscopy. For the detection by flow cytometry, EPCs were digested with 0.25% trypsin and permeabilized with 0.1% Triton-X at 37°C for 30 min. Subsequently, these cells were incubated with antibodies against CD133, CD34, or VEGFR2 (1:50, Santa Cruz Biotechnology); detected by a CytoFLEX flow cytometer (Beckman Coulter); and analyzed with the CytExpert software.

### RNA Extraction and Quality Control

Total RNAs were isolated from EPCs using the Small RNA Purification Kit (EZB-RN3, China) following the manufacturer’s instructions. The quality and quantity of total RNAs were measured using the NanoDrop 2000 spectrophotometer (Thermo Fisher Scientific), and RNA integrity number (RIN) was generated using the Agilent 2,100 Bioanalyzer (Agilent Technologies). The samples with RIN >7.0 were subjected for small RNA sequencing.

### Small RNA Library Preparation and Sequencing

About 1 μg of total RNAs from each sample was used to create small RNA library using the Small RNA Sample Preparation Kit (Illumina, San Diego, CA, United States) according to the manufacturer’s protocols. Briefly, the 3 and 5′ end of RNAs were ligated by the adapters. Then, the adapter ligated RNAs were reversely transcribed into cDNAs. Polymerase chain reactions (PCRs) were used to amplify the libraries, and polyacrylamide gel electrophoresis was performed for size selection. Finally, the amplified cDNAs were purified, and the library quality was evaluated on the Agilent 2,100 Bioanalyzer. All libraries were sequenced on the Illumina sequencing platform (HiSeqTM 2,500).

### Data Analysis of Small RNA Sequencing

The redundant reads including the extraneous sequences, adaptor sequences, and low-quality sequences were removed from the raw data using Illumina CASAVA (version 1.8). The clean reads were obtained by discarding sequences shorter than 15 nt or longer than 35 nt using the cutadapt and fqtrim. Then, Bowtie software was used to index the genome. Then, the clean reads were aligned to the rat reference genome assembly using the miRDeep software. The remaining reads were used to identify the known miRNAs by comparing with the corresponding miRNA precursor sequences from miRBase. The read counts of the identified miRNA in each sample were generated, and only the ones with an overlap more than 50% of miRBase-defined miRNAs were considered significant. The analysis of differentially expressed miRNAs between the high glucose and control groups was performed using DESeq packages. The miRNAs with a nominal *p* value < 0.05 and a fold change >2 were identified as differentialy expressed.

### Gene Ontology and Kyoto Encyclopedia of Genes and Genomes Pathway Analysis of Dysregulated MiRNAs

To predict the possible biological function of differentially expressed miRNAs, the target genes were predicted by TargetScan (http://www.targetscan.org). The biological process (BP), cellular component (CC), molecular function (MF), and Kyoto Encyclopedia of Genes and Genomes (KEGG) pathway of the miRNAs were exhibited using the Gene Ontology (GO) project (http://www.geneontology.org) and the KEGG database (http://www.kegg.jp/), respectively. Fisher’s exact test was used to determine the significant GO terms and pathway categories (*p* < 0.05).

### Quantitative Reverse Transcription-PCR Validation

To verify the results of small sequencing, quantitative reverse transcription-polymerase chain reaction (qRT-PCR) was conducted to detect the expression pattern of 20 dysregulated miRNAs. The extracted RNAs were reversely transcribed into cDNAs using MicroRNA Reverse Transcription Kit (EZB-miRT4, China) with the specific stem-loop RT-primer, according to the manufacturer’s instructions. PCR products were amplified using the EZ-probe qPCR Master Mix for microRNA (EZB-miProbe-R2, China) on the PikoReal Real-Time PCR System (Thermo Fisher Scientific). The relative miRNA expression level was determined using U6 as an endogenous control and calculated with the 2^−ΔΔCt^ method. The primer sequence of miRNAs are shown in Supplementary Table S1.

### miR-375–3p Transfection

EPCs were grown to approximately 70% confluence and transfected with the miR-375–3p mimics, inhibitor, or the corresponding negative controls using Lipo 6,000 Transfection Reagent (C0526, Beyotime, China) according to the manufacturer’s protocols. The sequences of miR-375–3p mimics, inhibitor, and the negative controls are shown below: miR-375–3p mimics sense: 5′-UUU​GUU​CGU​UCG​GCU​CGC​GUG​A-3′, antisense: 5′-ACG​CGA​GCC​GAA​CGA​ACA​AAU​U-3′; miR-375–3p inhibitor: 5′-UCA​CGC​GAG​CCG​AAC​GAA​CAA​A-3′; negative control mimics sense: 5′-UUC​UCC​GAA​CGU​GUC​ACG​UTT-3′, antisense: 5′-ACG​UGA​CAC​GUU​CGG​AGA​ATT-3′; negative control inhibitor: 5′-CAG​UAC​UUU​UGU​GUA​GUA​CAA-3′. The transfection efficacy was examined by qRT-PCRs.

### MTT Assay

The viability of EPCs was detected by MTT assay. Briefly, EPCs were seeded onto 96-well plates at the density of 1.5 × 10^4^ cells per well. After the required treatment, EPCs were incubated with MTT (5 mg/ml) in darkness at 37°C for 3 h. Then, the culture medium was removed, and DMSO (100 μl/well) was added into each well to dissolve the formazan crystals. Finally, the absorbance was determined at 595 nm by a microplate reader (FilterMax F5, Molecular Devices).

### Cell Proliferation Assay

Cell proliferation was examined by CCK-8 assay (Dojindo, Kumamoto, Japan). Briefly, EPCs were plated onto a 96-well plate, and CCK-8 solution was added to each well. After incubation for 1 h at 37°C, the absorbance was determined at a 450-nm wavelength using a microplate reader (Molecular Devices).

### Apoptosis Assay

The apoptosis of EPCs was determined using the Annexin V-FITC/PI Apoptosis Detection Kit (Cat: A211-01, Vazyme). After the required treatment, EPCs were digested with trypsin without EDTA and washed with PBS. These cells were re-suspended in 100 μl binding buffer. Under dark condition, 5 μl of Annexin V-FITC and PI Staining Solution were added to the suspension. Then, 400 μl binding buffer was added after 20 min. Finally, these cells were detected by the CytoFLEX flow cytometer.

### Transwell Assay

Transwell assay was conducted to detect cell migration ability. After the required treatment, EPCs were seeded onto the top chamber with polycarbonate filters (8-μm pore size; Corning). They were cultured for 18 h at 37°C, and the cells that did not pass through the membrane pores were removed by cotton swabs. After fixation, the cells on the lower surface were stained with 0.5% crystal violet (C805211, Macklin) for 45 min. The migrating cells were photographed using a bright-field microscope, and five random fields were counted.

### Tube Formation Assay

Tube formation assay was performed to detect the angiogenic activity of EPCs *in vitro*. Briefly, EPCs were re-suspended with the complete EGM-2 medium after the required treatment. They were then seeded onto a 24-well plate coated with Matrigel (356,234, Corning) at a density of 4 × 10^4^/ml and incubated for 8 h at 37°C in 5% CO_2_. The tube formation ability of EPCs was examined under a light microscope (OLYMPUS, Japan), and the length of tube formation was quantified by Image J software.

### Clinical Sample Collection

DR patients were diagnosed according to the American Diabetes Association’s diagnostic criteria and fundus photography results. Patients with cancer, systemic diseases, and other ocular diseases were excluded. Non-diabetic cataract patients or non-diabetic patients with macular hole were included as the control group. Whole blood (5 ml) was collected from each patient. The blood was transferred to an anticoagulant tube and centrifuged at 3,000 × *g* for 10 min to collect the plasma fraction and cellular fraction. Aqueous humor samples were harvested from patients with DR and cataract during surgeries according to the Declaration of Helsinki. About 200 μl of aqueous humor was collected from each patient. Vitreous samples were collected from patients undergoing pars plana vitrectomy for the treatment of DR and macular hole for non-diabetic patients. Vitreous samples were collected, placed immediately on ice, centrifuged for 15 min to remove insoluble materials, and stored at 80°C until use. For miRNA detection, about 1 ml of plasma, 200 μl of aqueous humor, or 300 μl of vitreous samples were used for RNA isolation by the TRIzol^®^ Reagent (Invitrogen) according to the manufacturer’s instructions. First, the samples were centrifuged at 12,000 × *g* for 15 min at 4°C to obtain clear supernatant. Then, equal volumes of TRIzol reagents and glycogen were added to increase the extraction specificity. Subsequently, chloroform was added and incubated for 5 min. The mixture was centrifuged at 12,000 × *g* for 15 min at 4°C, and the upper aqueous phase was collected and mixed with ethanol. Finally, the supernatant was removed. The remaining RNA pellet was washed and dissolved in DEPC water.

### Statistical Analysis

All statistical analyses were performed using GraphPad Prism version 8.0. The difference was determined by Student *t* test (two-group comparison) or one-way ANOVA followed by the *post hoc* Bonferroni test (multi-group comparison). *p* < 0.05 was considered significant.

## Results

### Characterization of BM-Derived EPCs

**FIGURE 1:**
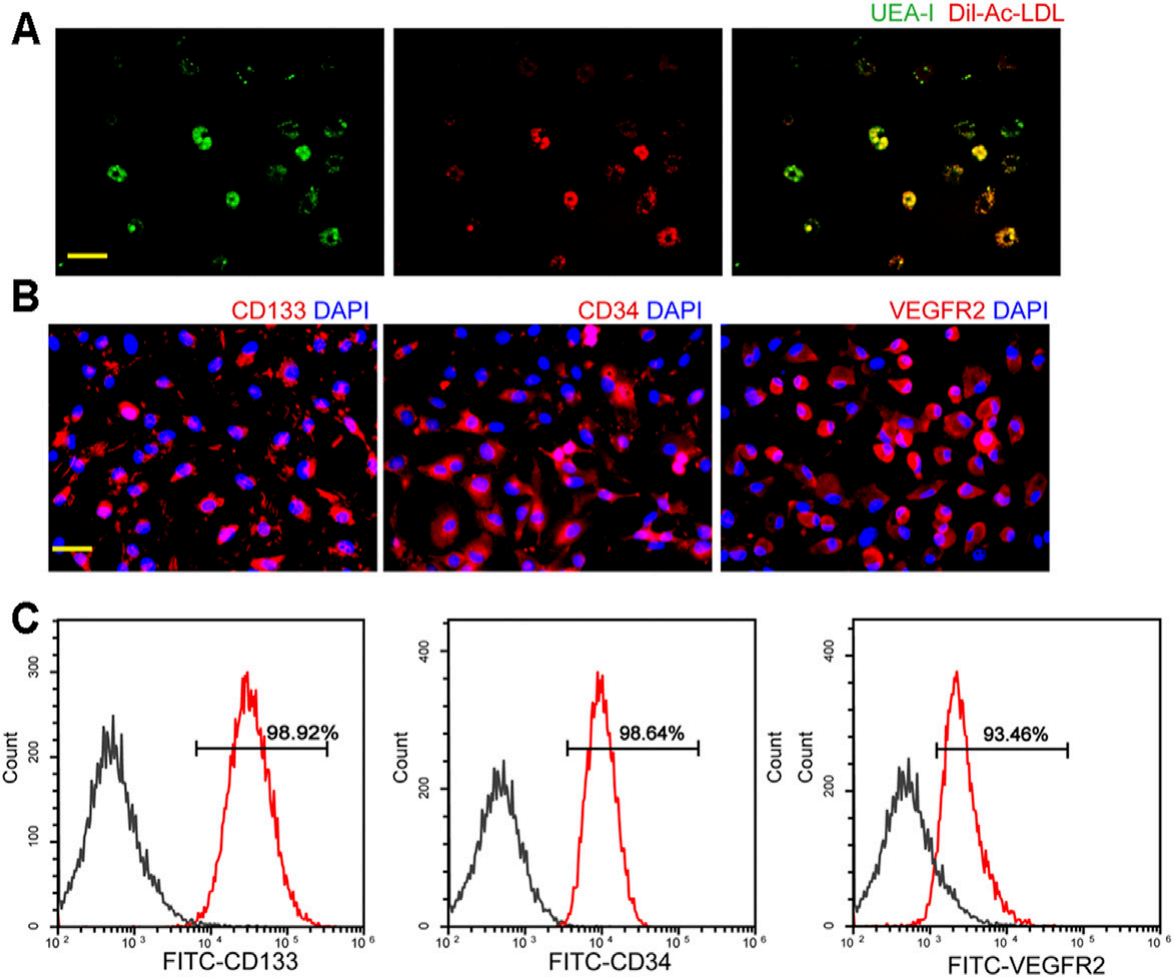
Characterization of endothelial progenitor cells (EPCs). **(A)** Staining of Dil-conjugated acetylated low-density lipoprotein (Dil-Ac-LDL), FITC-Ulex Europaeus Agglutinin I (UEA-1), and merged images of double staining of Dil-Ac-LDL and FITC-UEA-I. Scale bar: 20 μm. **(B)** Immunofluorescent staining demonstrated that EPCs had a positive expression of CD133, CD34, and VEGFR2. Scale bar: 20 μm. **(C)** Flow cytometry analysis showed the expressions of CD133, CD34, and VEGFR2 in EPCs. All experiment was repeated three times.

To identify the EPCs, double-positive staining, immunofluorescent staining, and flow cytometry were conducted. Double staining with the functional markers, UEA-l and Dil-Ac-LDL, indicated that the isolated cells were EPCs (Figure 1A). To further characterize EPCs, the expression of CD133, CD34, and VEGFR2 was examined using immunofluorescent staining and flow cytometry. As shown in Figure 1B, the majority of the isolated EPCs expressed surface markers, including CD133, CD34, and VEGFR2. Flow cytometry assays showed 98.92% of the isolated cells were CD133-positive, 98.64% of the isolated cells were CD34-positive, and 93.46% of the isolated cells were VEGFR2-positive, suggesting that the majority of the isolated cells were EPCs (Figure 1C).

### Analysis of MiRNA Expression Profiling of EPCs Following High Glucose Stress

**FIGURE 2:**
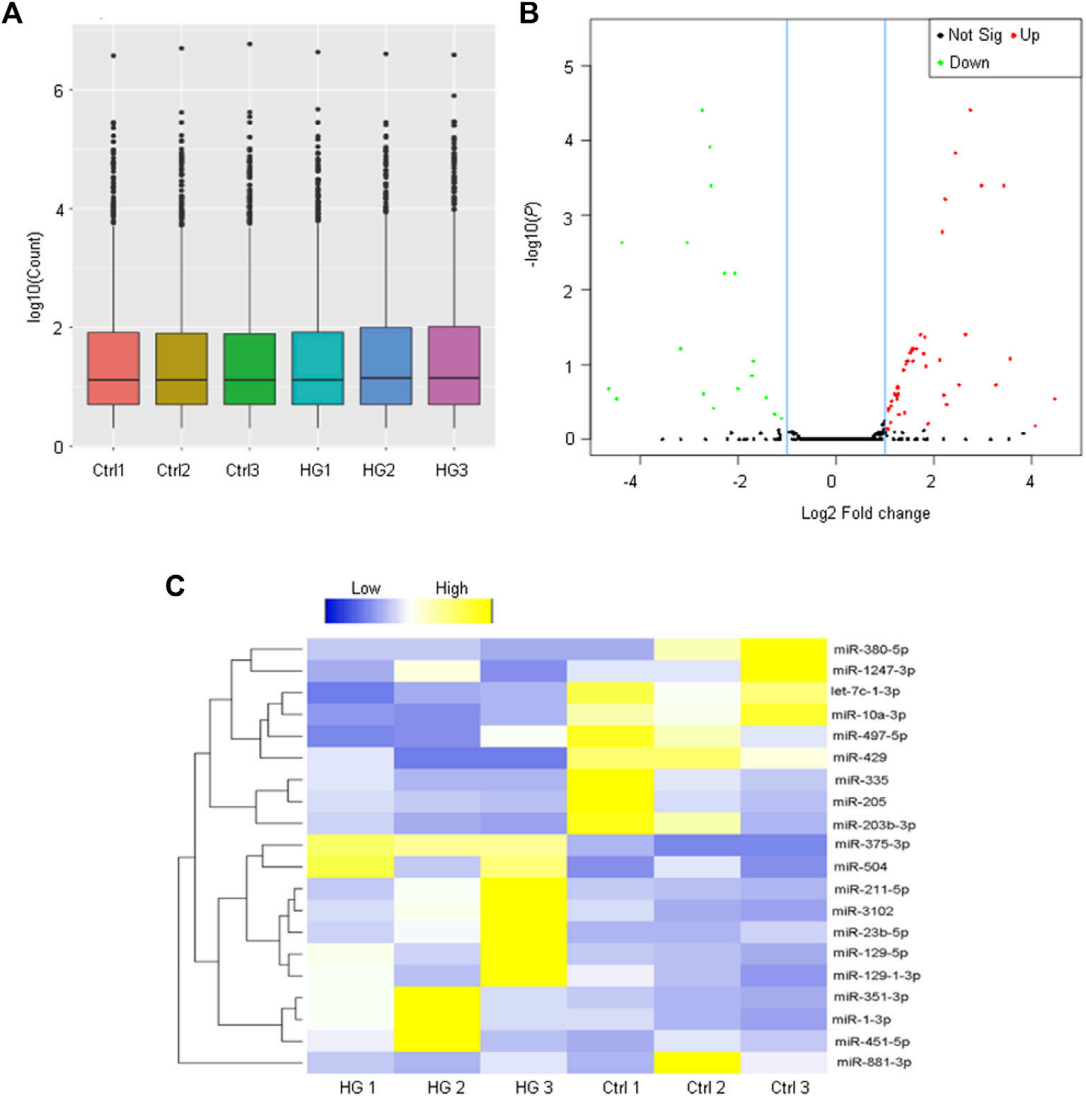
Analysis of miRNA profiling of EPCs derived from the bone marrow. **(A)** The box plot is depicted to show the distribution of miRNA expression pattern across different samples. The boxes with a central line and two tails represent the median of the data and the upper and lower quartiles, respectively. **(B)** The volcano plot shows the differentially expressed miRNAs between EPCs derived from the bone marrow with or without high-glucose treatment. **(C)** Heatmaps generated from hierarchical cluster analysis display 20 differentially expressed miRNAs between EPCs derived from the bone marrow with or without high-glucose treatment. Yellow color and blue color indicate the up-regulated and down-regulated miRNAs, respectively.

To investigate the potential functions of miRNAs in EPCs, we investigated the miRNA expression profiles of EPCs derived from the bone marrow with or without high glucose stress. A box plot demonstrated that the distributions of miRNA expression datasets across different samples are not significantly different after normalization (Figure 2A), indicating that miRNA expression change was not random. A volcano plot was conducted to identify the differentially expressed miRNAs between the paired two groups (Figure 2B). A total of 72 miRNAs were differentially expressed with fold change greater than 2 and *p* < 0.05, including 53 up-regulated and 19 down-regulated miRNAs between normal EPCs and high glucose-treated EPCs (Supplementary Table S2). To demonstrate the expression patterns of significant miRNAs, we built up a hierarchically clustered heatmap using the top 10 up-regulated miRNAs and the top 10 down-regulated. The differentially expressed miRNAs were segregated into two groups (Figure 2C).

### Validation for the Results of Small RNA Sequencing

To validate the results of small RNA sequencing, qRT-PCRs were conducted to detect the expression pattern of 20 differentially expressed miRNAs, including 10 up-regulated miRNAs and 10 down-regulated miRNAs. We found a general consistency between qRT-PCR results and small RNA sequencing results (Table 1). Eight of the 10 up-regulated miRNAs and 8 of the 10 down-regulated miRNAs exhibited similar expression patterns as a result of small RNA sequencing.

**TABLE 1:** Verification of miRNA sequencing results by qRT-PCRs.

	Log_2_FC			
miRNA Id	RNA sequence	Q-PCR		Verified
rno-miR-6321	4.59	1.12	Up-regulated	Yes
rno-miR-293–5p	4.47	−1.08		No
rno-miR-299a-5p	4.08	−1.97		No
rno-miR-96–5p	3.56	3.54	Up-regulated	Yes
rno-miR-490–5p	3.44	2.13	Up-regulated	Yes
rno-miR-375–3p	3.27	4.35	Up-regulated	Yes
rno-miR-210–5p	2.98	2.93	Up-regulated	Yes
rno-miR-490–3p	2.75	4.19	Up-regulated	Yes
rno-miR-144–3p	2.65	2.42	Up-regulated	Yes
rno-miR-466b-2-3p	2.52	1.90	Up-regulated	Yes
rno-miR-204–3p	−4.65	−1.33	Down-regulated	Yes
rno-miR-463–3p	−4.49	−1.85	Down-regulated	Yes
rno-miR-672–3p	−4.49	−1.96	Down-regulated	Yes
rno-miR-743b-5p	−4.38	1.25		No
rno-miR-743a-5p	−3.18	−2.27	Down-regulated	Yes
rno-miR-743b-3p	−3.05	−2.78	Down-regulated	Yes
rno-miR-203b-3p	−2.73	−1.30	Down-regulated	Yes
rno-miR-380–5p	−2.71	2.80		No
rno-miR-335	−2.58	−4.55	Down-regulated	Yes
rno-miR-205	−2.55	−2.08	Down-regulated	Yes

### GO and KEGG Pathway Analysis of the Putative Target Genes of Differentially Expressed MiRNAs

**FIGURE 3:**
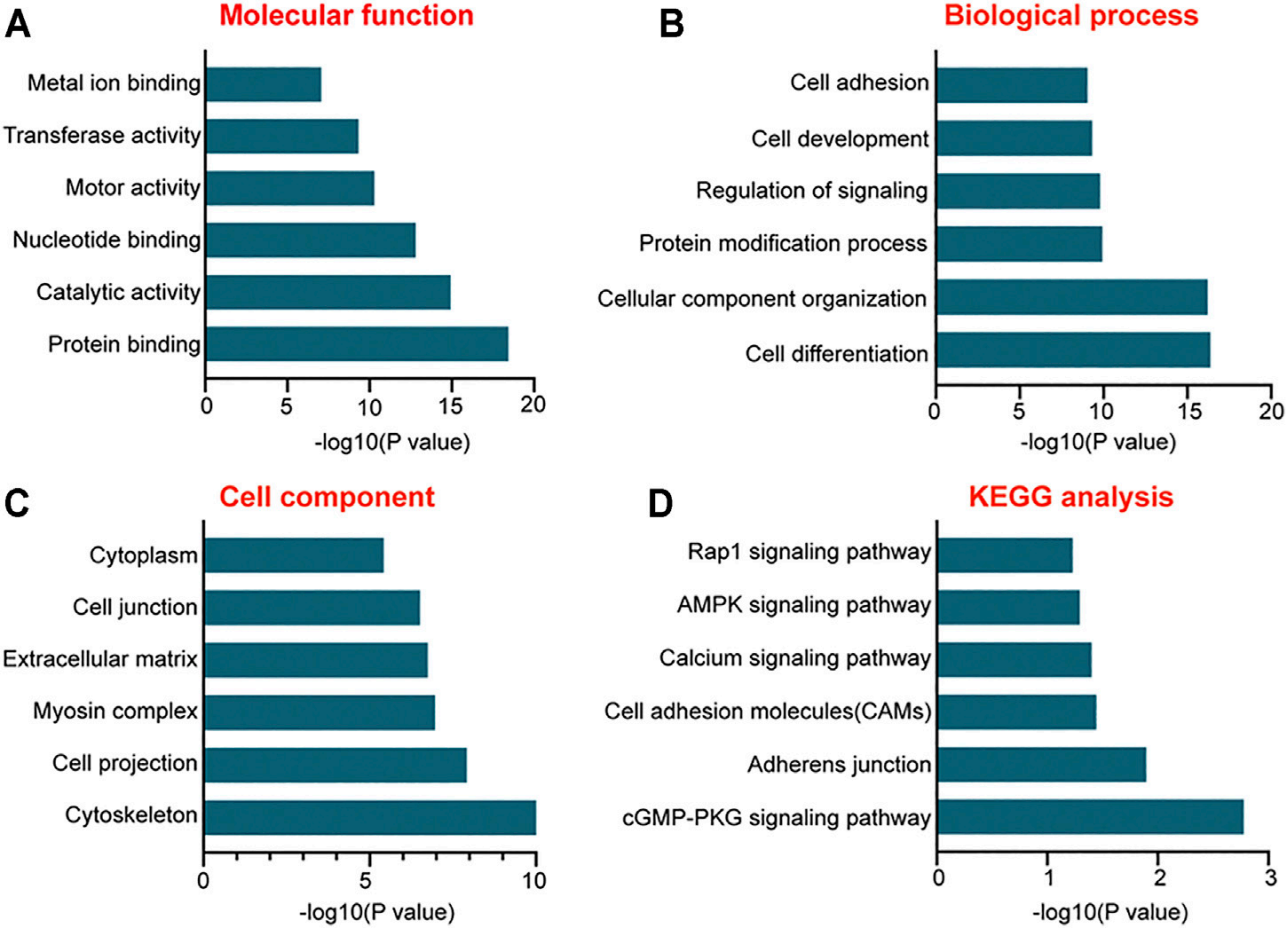
Gene Ontology (GO) and Kyoto Encyclopedia of Genes and Genomes (KEGG) pathway analysis of the putative target genes of differentially expressed miRNAs. **(A–C)** The functions of the putative target genes of differentially expressed miRNAs in EPCs using GO analysis. The GO enrichment was divided into three parts: molecular function **(A)**, biologic process **(B)**, and cellular component **(C,D)** The bar plot shows the results of KEGG pathway analysis and reveals the top six signaling pathways involved in miRNA-mediated regulatory network.

To further probe into the functions of these differentially expressed miRNAs, GO enrichment and KEGG pathway analyses were conducted. GO analysis based on the putative target genes of miRNAs was divided into three subgroups: biological process, molecular function, and cellular component (Figures 3A–C). In terms of the biological process, the most significantly enriched GO term was “cell differentiation” and the most enriched GO term in the molecular function was “protein binding.” As to the cellular component, GO term was tightly associated with “cytoskeleton.” Pathway analysis was conducted to map the target genes of differentially expressed miRNAs to KEGG pathways, which displayed the top six signaling pathways. Among them, cGMP-PKG signaling was the most enriched signaling pathway (Figure 3D).

### The Involvement of miR-375–3p in EPC Angiogenic Activity *In Vitro*

**FIGURE 4:**
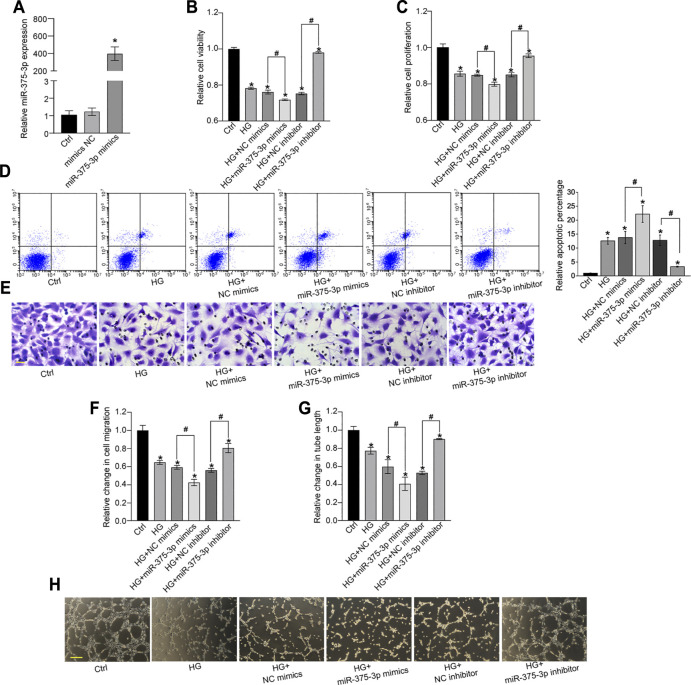
miR-375–3p regulates EPC function *in vitro*. **(A)** EPCs were transfected with negative control (NC) mimics, miR-375–3p mimics, or left untreated (Ctrl) for 24 h. The expression of miR-375–3p was detected by quantitative reverse transcription-polymerase chain reaction (qRT-PCR) **(A)**. **(B–H)** EPCs were transfected with NC mimics, miR-375–3p mimics, NC inhibitor, miR-375–3p inhibitor, or left untreated (Ctrl) for 24 h and then treated with or without high glucose (30 mM) for 24 h. The viability of EPCs was determined by MTT assays **(B)**. The proliferation ability of EPCs was determined by CCK-8 assays **(C)**. Flow cytometry assays and quantitative analysis were conducted to detect cell apoptosis **(D)**. Cell migration and quantitative analysis was conducted using Transwell assays [**(E,F)**, scale bar: 20 μm]. Tube formation assays and quantitative analysis were conducted to detect the tube-formation activity of EPCs [**(G,H)**, scale bar: 50 μm]. *p < 0.05 versus Ctrl group; Pound sign (^#^) indicates significant differences between the marked groups. All experiment was repeated three times.

miR-375–3p was shown to be significantly up-regulated in EPCs following high-glucose stress. Moreover, rno-miR-375–3p has a homologous gene in human genome with 100% similarity of gene sequence, hsa-miR-375–3p (Supplementary Figure S1). Transfection of miR-375–3p mimics led to increased levels of miR-375–3p expression in EPCs (Figure 4A). MTT assays revealed that transfection of miR-375–3p mimics led to decreased viability of EPCs (Figure 4B). CCK-8 assays showed that miR-375–3p mimics significantly decreased the proliferation ability of EPCs (Figure 4C). Flow cytometry assays showed that transfection of miR-375–3p mimics accelerated high glucose-induced apoptosis of EPCs as shown by the increased number of apoptotic cells (Figure 4D). By contrast, transfection of miR-375–3p inhibitor led to increased cell viability, increased proliferation ability, and reduced apoptotic number of EPCs (Figures 4B–D). The migration ability of EPCs was then determined using the Transwell assay. Transfection of miR-375–3p mimics led to decreased migration ability of EPCs, whereas transfection of miR-375–3p inhibitor led to increased migration ability (Figures 4E,F). We also conducted tube formation assay to determine the role of miR-375–3p on the tube formation ability of EPCs. The results showed that transfection of miR-375–3p mimics significantly reduced the number of branch points and tube length. By contrast, transfection of miR-375–3p inhibitor contributed to tube formation of EPCs (Figures 4G,H). We also established the hypoxic stress model *in vitro* by exposing EPCs to CoCl_2_ and exploring the role of miR-375–3p in EPC biology under hypoxic stress. Similarly, we also revealed that miR-375–3p inhibitor increased the viability and proliferation ability of EPCs, decreased CoCl_2_-induced cell apoptosis, and increased the migration ability and angiogenic ability of EPCs (Supplementary Figure S2).

### The Level of miR-375–3p Expression Is Up-Regulated in the Clinical Samples of DR Patients

**FIGURE 5:**
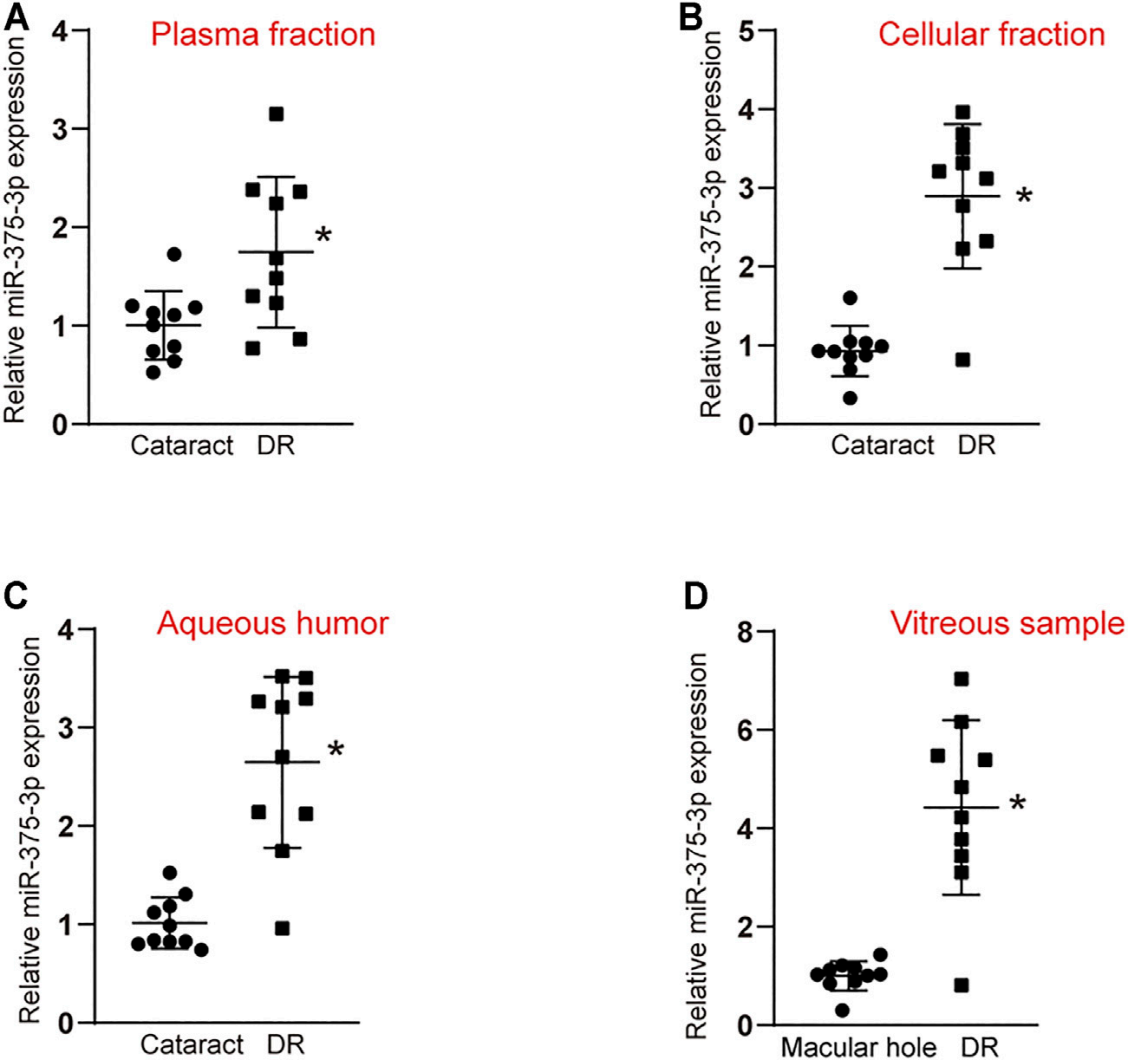
Evaluation of the diagnostic performance of miR-375–3p in diabetic retinopathy. **(A,B)** qRT-PCR assays were conducted to detect miR-375–3p expression in the plasma fraction **(A)** and cellular fraction **(B)** of peripheral blood of DR patients and cataract patients (*p < 0.05 vs. cataract, two-tailed Student *t* test). **(C)** qRT-PCR assays were conducted to detect miR-375–3p expression in aqueous humor of DR patients and cataract patients (*p < 0.05, two-tailed Student *t* test and Bonferroni test). **(D)** qRT-PCR assays were conducted to detect miR-375–3p expression in the vitreous samples of DR patients and non-diabetic patients with macular hole (*p < 0.05, two-tailed Student *t* test and Bonferroni test).

To explore the diagnostic value of miR-375–3p, we collected peripheral blood and aqueous humor from patients with DR and patients with cataract to detect the level of miR-375–3p expression. The results showed that the level of miR-375–3p expression was significantly up-regulated in the plasma fraction and cellular fraction of peripheral blood in DR patients (Figures 5A,B). Moreover, the level of miR-375–3p expression was significantly up-regulated in the aqueous humor of DR patients (Figure 5C). We also compared the expression pattern of miR-375–3p between non-diabetic patients with macular holes and patients with DR. qRT-PCRs showed that the level of miR-375–3p expression was significantly up-regulated in the vitreous samples of DR patients (Figure 5D). Collectively, these results suggest that miR-375–3p is a potential biomarker for the diagnosis of DR.

## Discussion

Endothelial dysfunction is the pathological basis of DR and a risk factor for retinal vascular dysfunction (Franssen et al., 2016; Zhang et al., 2020). However, the repair ability of mature endothelial cells is very limited. Many researches have uncovered EPCs as the precursor cells for mature endothelial cells, which are involved in angiogenesis, endothelium repair, and vasculogenesis (Wang et al., 2018). Previous studies have shown that the numbers and functions of EPCs were damaged in DR, which can delay endothelial repair and exacerbate vascular injury (Desouza et al., 2011; Zhang and Yan 2013). MiRNAs are potential regulators of EPC biology, and intervention of miRNA expression may affect the development of vascular function by targeting EPCs in several diseases, such as deep vein thrombosis and cerebral infarction (Qu et al., 2015; Wang et al., 2018; Sun et al., 2019). In this study, we performed small RNA sequencing and identified EPC dysfunction-related miRNAs following high-glucose stress. A total of 72 miRNAs were significantly differentially expressed between the high glucose-treated group and the control group, including 53 up-regulated and 19 down-regulated miRNAs. This study provides a theoretical basis for further investigation of miRNA function in EPC biology.

It has been observed that dysregulated expression of miRNAs participates in the pathogenesis of vascular diseases (Saliminejad et al., 2019; Farina et al., 2020). The main function of miRNA is negative regulation of gene expression by binding the target mRNAs and induction of the degradation or inhibition of translation (Krol et al., 2010; Beermann et al., 2016). Hence, it is reasonable to speculate the functions of miRNAs *via* the functions of their target genes. According to the results of GO enrichment analysis, we found that “protein binding,” “cell differentiation,” and “cytoskeleton” are the most significant enriched GO terms of the target genes of dysregulated miRNAs. miRNAs are primarily located in cytoplasm, act as post-transcriptional regulators by binding with Ago 2 protein, and then alter gene expression (Ameres and Zamore 2013). Under hyperglycemia stress, the dysregulated miRNAs might affect EPC functions *via* regulating cell cytoskeleton, mysion complex, and extracellular matrix components, which can involve in the process of cell differentiation, cell development, and cell adhesion. Eventually, the repair progression of endothelial cells can be interfered, aggravating the occurrence of vascular injury in DR. KEGG pathway analysis revealed that the cGMP-PKG signaling pathway is mainly involved in miRNA-mediated mechanism. Notably, cGMP-PKG signaling has been reported to be involved in the pathogenesis of vascular dysfunction and tissue regeneration (Franssen et al., 2016; Meakin et al., 2020; Schall et al., 2020). Based on the aforementioned evidence, we postulate that miRNA-mediated regulatory network is involved in the regulation of EC function in DR.

EPCs have been shown as a useful biomarker for cardiovascular diseases and cancers. Based on the EPC number and function, it is possible to predict whether individuals have a great risk for retinal vascular dysfunction in DR. We show that miR-375–3p is a critical regulator of EPC biology. Inhibition of miR-375–3p protects against hyperglycemic stress- or hypoxic stress-induced EPC injury as shown by increased viability, proliferation, migration, and tube formation ability of EPCs and decreased apoptosis. MiRNAs are highly conserved in eukaryotic transcriptome and are expressed in a tissue-specific pattern (Lee et al., 2004; Saliminejad et al., 2019). Many miRNAs have already successfully been shown to serve as biomarkers or therapeutic targets for different diseases (Beermann et al., 2016). The levels of miR-375–3p expression are significantly up-regulated in aqueous humor, vitreous samples, and peripheral blood of patients with DR. Thus, it is possible to use EPCs as cell biomarkers and use miR-375–3p as a molecular marker for the diagnosis of DR (Medina et al., 2010; Yu et al., 2016).

EPCs are essential for maintaining retinal vessel integrity and homeostasis (Lois et al., 2014). Accumulating evidence suggests that the number and function of EPCs are altered with varied diabetes duration, metabolic control, and in the presence or absence of DR. These isolated EPCs show dysfunctional responses including impaired vasoreparative potential and premature senescence. The recovery of the number and function of EPCs could underpin a lesser risk of DR progression. We identify miR-375–3p as a key regulator of EPC function. Thus, intervention at miR-375–3p level is a therapeutic method for the recovery of EPC dysfunction. In many clinical trials, anti-VEGF drugs (ranibizumab, bevacizumab, and aflibercept) have demonstrated their safety even when administered monthly (Elman et al., 2010; Mitchell et al., 2011; Do et al., 2012). Local delivery into the eye by means of intravitreal injections of EPCs would be an option for the treatment of EPC dysfunction. In addition, considering the fact that DM is a systemic disorder in which other organs besides the eye are affected, systemic administration should not be ruled out. Systemic administration of EPC transplant or systemic EPC mobilization also contributes to the treatment of diabetes-induced retinal vascular dysfunction (Jørgensen et al., 2014; Lois et al., 2014).

Taken together, our study revealed the overall expression profiles of miRNAs in EPCs under hyperglycemia and identified miR-375–3p as a biomarker for diabetic microvascular disease. We also preliminarily investigated the role of miR-375–3p in EPC biology. In the future, we will focus on the study of its mechanism to investigate the significance of dysregulated miRNAs in the pathogenesis of EPC dysfunction.

## Data Availability

The original contributions presented in the study are publicly available. This data can be found here: National Center for Biotechnology Information (NCBI) BioProject database under accession number PRJNA770901.
