# Supplementary material for: Differential MicroRNA Expression Pattern in Endothelial Progenitor Cells During Diabetic Retinopathy
Source: Front Cell Dev Biol. 2021 Dec 16;9:773050. doi: 10.3389/fcell.2021.773050 (PMC8717624; doi:10.3389/fcell.2021.773050)
Supplement: Supplementary file 1 [file Presentation1.PDF]

Supplementary Figure 1: Homologous gene comparison of miR-375-3p

BLAST was performed to compare the sequence similarity of miR-375-3p between human genome and rat genome.

```
# Aligned_sequences: 2
# 1: rno-miR-375-3p
# 2: hsa-miR-375-3p
# Matrix: EDNAFULL
# Gap_penalty: 10.0
# Extend_penalty: 0.5
#
# Length: 22
# Identity:      22/22 (100.0%)
# Similarity:    22/22 (100.0%)
# Gaps:           0/22 ( 0.0%)
# Score: 110.0
#
#
#=======================================

rno-miR-375-3      1 UUUGUUCGUUCGGCUCGCGUGA     22
                     ||||||||||||||||||||||
hsa-miR-375-3      1 UUUGUUCGUUCGGCUCGCGUGA     22


#---------------------------------------
#---------------------------------------
```

Supplementary Figure 2: miR-375-3p regulates EPCs function under hypoxic stress

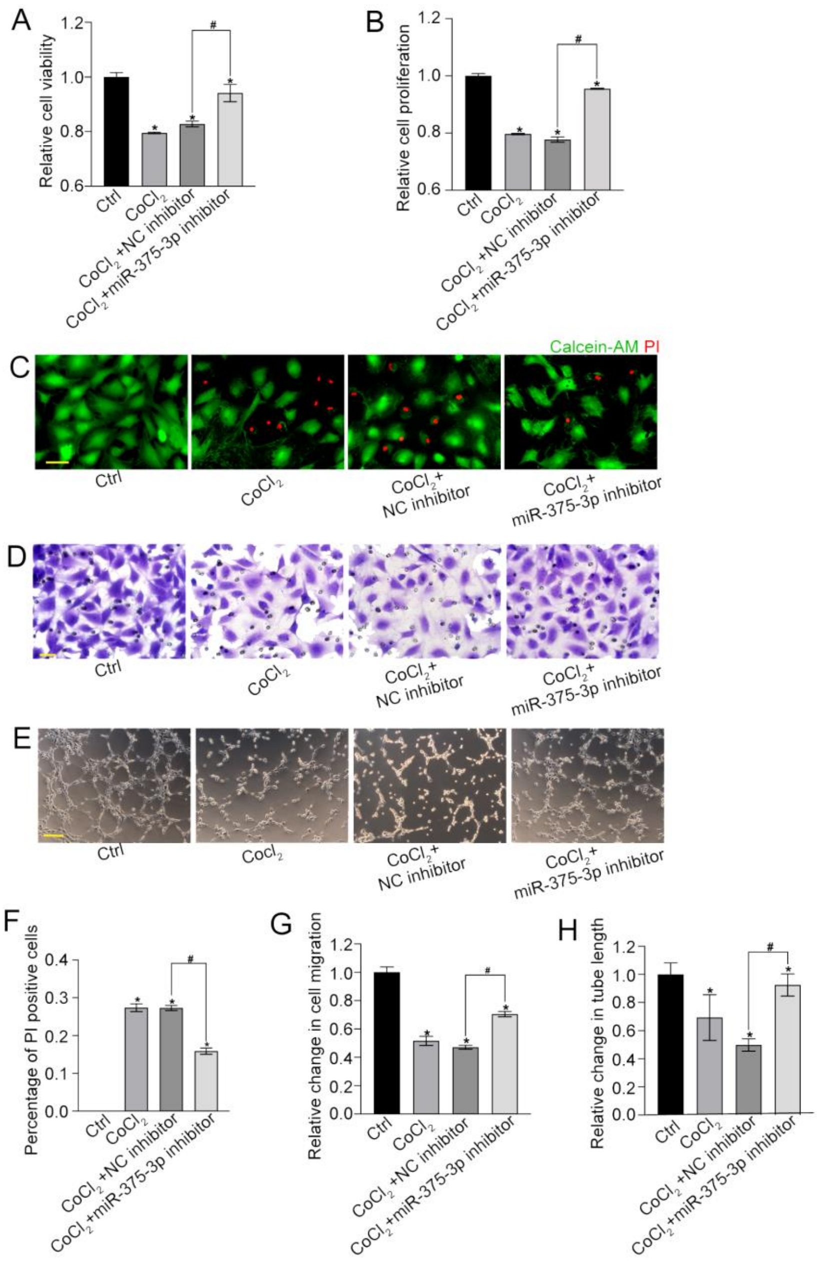

EPCs were transfected with negative control (NC) inhibitor, miR-375-3p inhibitor, or left untreated (Ctrl) for 24 h, and then treated with or without $CoCl_2$ (200 μmol/L) for 24 h. The viability of EPCs were determined by MTT assays (A, n=3). The proliferation ability of EPCs were measured by CCK-8 assays (B, n=3). Calcein-AM/PI double staining assays and quantitative analysis were conducted to detect cell apoptosis (C and F, n=3, Scale bar: 20 μm). Cell migration and quantitative analysis was conducted using Transwell assays (D and G, n=3, Scale bar: 20 μm). Tube formation assays and quantitative analysis were conducted to detect the tube formation activity of EPCs (E and H, n=3, Scale bar: 50 μm). $^*P < 0.05$ versus Ctrl group; "#" indicated significant differences between the marked groups.
